# *Hox10* Genes Function in Kidney Development in the Differentiation and Integration of the Cortical Stroma

**DOI:** 10.1371/journal.pone.0023410

**Published:** 2011-08-16

**Authors:** Alisha R. Yallowitz, Steven M. Hrycaj, Kieran M. Short, Ian M. Smyth, Deneen M. Wellik

**Affiliations:** 1 Department of Internal Medicine, Division of Molecular Medicine and Genetics University of Michigan, Ann Arbor, Michigan, United States of America; 2 Department of Biochemistry and Molecular Biology, Monash University, Clayton Campus, Melbourne, Victoria, Australia; 3 Department of Anatomy and Developmental Biology, Monash University, Clayton Campus, Melbourne, Victoria, Australia; The University of Hong Kong, China

## Abstract

Organogenesis requires the differentiation and integration of distinct populations of cells to form a functional organ. In the kidney, reciprocal interactions between the ureter and the nephrogenic mesenchyme are required for organ formation. Additionally, the differentiation and integration of stromal cells are also necessary for the proper development of this organ. Much remains to be understood regarding the origin of cortical stromal cells and the pathways involved in their formation and function. By generating triple mutants in the *Hox10* paralogous group genes, we demonstrate that *Hox10* genes play a critical role in the developing kidney. Careful examination of control kidneys show that *Foxd1-*expressing stromal precursor cells are first observed in a cap-like pattern anterior to the metanephric mesenchyme and these cells subsequently integrate posteriorly into the kidney periphery as development proceeds. While the initial cap-like pattern of *Foxd1*-expressing cortical stromal cells is unaffected in *Hox10* mutants, these cells fail to become properly integrated into the kidney, and do not differentiate to form the kidney capsule. Consistent with loss of cortical stromal cell function, *Hox10* mutant kidneys display reduced and aberrant ureter branching, decreased nephrogenesis. These data therefore provide critical novel insights into the cellular and genetic mechanisms governing cortical cell development during kidney organogenesis. These results, combined with previous evidence demonstrating that *Hox11* genes are necessary for patterning the metanephric mesenchyme, support a model whereby distinct populations in the nephrogenic cord are regulated by unique *Hox* codes, and that differential *Hox* function along the AP axis of the nephrogenic cord is critical for the differentiation and integration of these cell types during kidney organogenesis.

## Introduction

Metanephric kidney development initiates at approximately E10.5 in mice, when the metanephric mesenchyme condenses at the posterior end of the nephrogenic cord adjacent to the hindlimbs. These cells signal to the Wolffian duct to promote evagination of the ureteric bud, which invades the metanephric mesenchyme. Subsequent reciprocal inductive interactions between the nephrogenic mesenchyme and the ureter ultimately lead to the formation of the mature branched kidney [1], [2], [3]. Initially, *Gdnf* expression in the metanephric mesenchyme is recognized by the receptors *Ret* and Gfrα1 on the ureteric epithelium and causes both the formation and invasion of the UB into the mesenchyme [4], [5], [6], [7], [8], [9], [10], [11], [12], [13], [14]. After UB invasion, a number of transcription factors including *Pax2*, *Eya1*, *Wt1*, *Sall1*, and the *Hox11* are necessary to maintain *Gdnf* expression in the mesenchyme, promote continued proliferation and expansion of the mesenchyme and to control the further budding and branching of the epithelial ureteric tree [15], [16], [17], [18], [19], [20], [21], [22], [23]. In turn, the ureteric tree expresses *Fgf2* and *Bmp7* to promote survival and proliferation of the mesenchyme and *Wnt9b* to initiate the formation of cap mesenchyme around the branch tips [24], [25], [26], [27], [28]. Undifferentiated cap mesenchyme expresses *Six2*, which regulates nephrogenic progenitor cell renewal [29]. Finally, a portion of the cap mesenchyme expresses *Wnt4* and subsequently undergoes epithelialization to form nephrons [30], [31].

In addition to the nephrogenic mesenchyme and ureter epithelium, a third cell type is present during early kidney organogenesis, the stromal cell population. Stromal cells are first observed just after ureteric bud invasion as a group of cells that surround the condensed mesenchyme [32]. A number of genetic studies have shown that stromal cells play critical roles during kidney organogenesis including producing signals that maintain outer and inner zones of differentiation, regulating pathways involved in the differentiation of nephrons, ureter branching morphogenesis, and in regulating the formation of the kidney capsule [32], [33], [34], [35], [36], [37], [38], [39]. At present, the transcription factor *Foxd1* is the earliest known marker of stromal cells and is exclusively expressed in these cells as early as E11.5 [34]. Functional analyses have shown that the loss of *Foxd1* results in a reduction of nephron number, defects in ureter branching and loss of kidney capsule formation [34], [36]. However, much remains to be determined regarding the origin of stromal cells, how they become integrated into the kidney and the signaling networks involved in their function.

*Hox* genes are expressed along the anterior-posterior (AP) body axis and are necessary for patterning many mesodermal organs. While 28 of the 39 *Hox* genes are expressed in the kidney [40], only the *Hox11* paralogous group genes have been shown to play functional roles during mammalian kidney development [18], [22]. Prior to UB invasion, the *Hox11* genes are expressed in the condensed metanephric mesenchyme and then later become localized to the nephrogenic cap mesenchyme as nephrogenesis proceeds [41], [42], [43]. Functional studies have further shown that *Hox11* proteins activate *Six2* and *Gdnf* expression in the metanephric mesenchyme [18], [22], [44]. As a result of this regulatory role, the UB fails to form in *Hox11* mutant mice and the metanephric mesenchyme subsequently undergoes apoptosis [22].

*Hox10* genes are also strongly expressed in the developing kidney at multiple stages of embryogenesis. Interestingly, while the expression patterns of both *Hox10* and *Hox11* are largely overlapping, we observe that the anterior boundary of *Hox10* expression in the nephrogenic cord is anterior to that of *Hox11*. Both *Hox10* and *Hox11* are similarly expressed in the nephrogenic mesenchyme at E13.5, however, we find that *Hox10* exhibits additional expression in the cortical stromal cells and suggests that these genes may be playing a unique role in the stroma. The functional data reported herein confirms this novel role and shows that the cortical stromal cells fail to properly differentiate and integrate into the kidney in *Hox10* mutants. This cellular defect results in aberrant ureter branching, decreased nephrogenesis and a loss of kidney capsule formation, phenotypes reminiscent of those reported for *Foxd1* [34], [36]. In summary, our results indicate that the *Hox10* genes play a critical role in regulating cortical stromal cell differentiation and integration in the mammalian kidney.

## Results

### Kidney morphology in *Hox10* mutants

There are three *Hox10* paralogs in mammals, *Hoxa10, Hoxc10* and *Hoxd10*. While a previous study has shown that *Hox10* triple mutants develop severe skeletal defects [45], the potential role(s) these genes play in kidney organogenesis has not been reported. In order to address this, mice with combinations of null mutations for *Hoxa10*, *Hoxc10*, and *Hoxd10* were generated. Our data indicate that mice with any combination of three mutant alleles within the *Hox10* paralogous group have no discernable kidney phenotype. This lack of mutant phenotype is likely due to the functional redundancy that exists among paralogous groups [22], [45], [46], [47], [48], [49], [50]. Consistent with this, combinations of four mutant alleles results in kidney abnormalities and these animals die two to six months after birth. Five-allele mutant mice demonstrate stronger defects and die between zero and eight weeks of age. Finally, *Hox10* triple mutants display the most severe phenotypes and die within 24 hours of birth.

**Figure 1:**
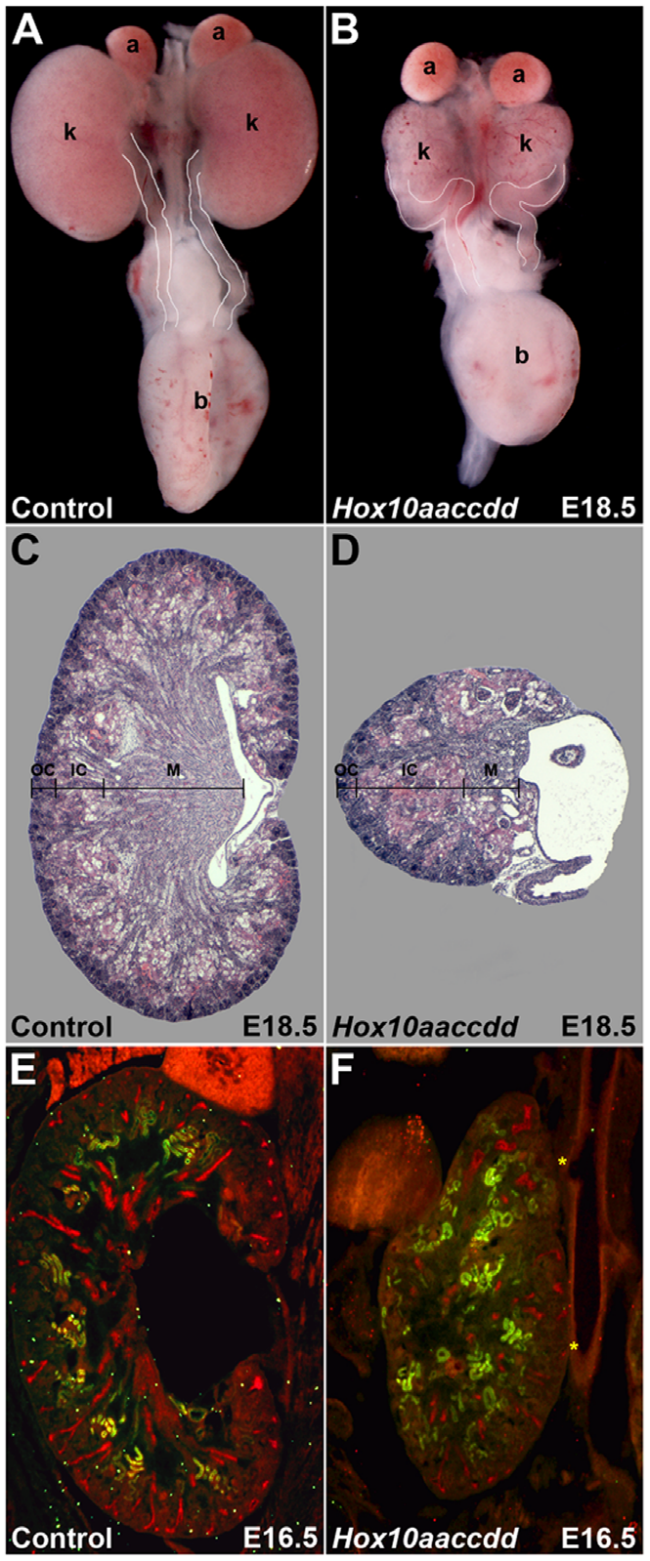
Kidney defects in Hox10 paralogous mutants. At E18.5, the kidneys from *Hox10* triple mutants (B) are much smaller than controls (A), and the ureter routing (white outline) is abnormal with the ureter entering the kidney from the lateral side, compared to a medial entry for controls. (C) Histological section through an E18.5 control kidney demonstrates organization of this stage kidney into three distinct regions, the outer cortex, OC, inner cortex, IC, and medulla, M. The zone of nephrogenesis is in the outer cortex and surrounds the kidney up to the point of ureter entry. (D) Histological section through an E18.5 *Hox10* triple mutant embryo shows a reduced nephrogenic zone in the outer cortex, an expanded inner cortex, a very reduced medullary region as well as hydronephrosis at the ureter-pelvic junction. (E and F). Frontal sections of E16.5 control (E) and *Hox10* mutant (F) embryos stained with LTL (green) to label proximal tubules and DBA (red) to label collecting ducts. Mature collecting duct structures do not develop properly and proximal tubules are not localized in a normal pattern in *Hox10* mutants (F). Yellow asterisks in (F) indicate fusions to the body wall. Embryos in (E) and (F) were sectioned through the same (frontal) plane. k, kidney; a, adrenal; b, bladder; OC, outer cortex; IC, inner cortex; M, medulla.

**Figure 2:**
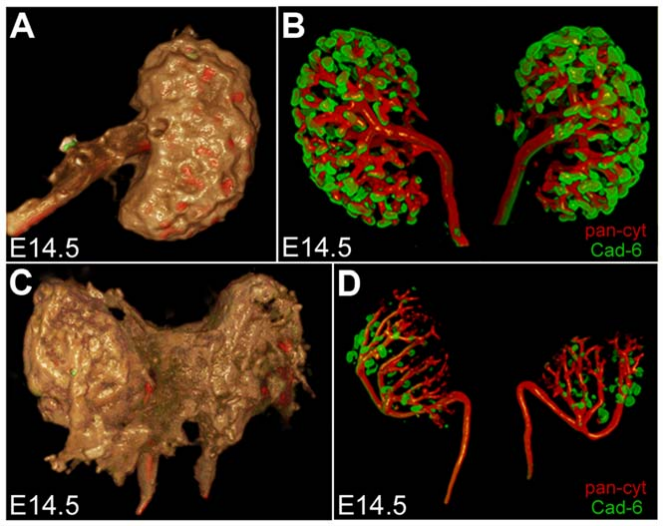
*Hox10* mutants exhibit ureter branching defects and kidneys do not detach from the body wall and urogenital ridge. (A–D) Visualization of the urogenital systems from E14.5 control (A, B) and *Hox10* mutant (C, D) embryos by OPT. Embryos were stained with *Pax2* (red) to label the nephrogenic mesenchyme and pan-cytokeratin (green) to label the epithelial structures. Overlay of background fluorescence delineates the organ surface (A, C brown), highlighting failure of the *Hox10* mutant kidney to separate from the body wall. Ureter branching in *Hox10* mutants (D) is reduced and branches are abnormally elongated compared to control animals (B).

Overtly, *Hox10* triple mutant kidneys are hypoplastic and morphologically underdeveloped as compared to controls (Fig. 1). Histologically, H/E staining at E18.5 reveals that the outer cortex of *Hox10* mutant kidneys extends around only a portion of the kidney, the inner cortex is expanded, and the medulla is greatly reduced (Fig. 1C, D). Notably, the medio-lateral position of the kidneys is reversed, which results in the mispositioning of the ureter-pelvic junction (UPJ) to the lateral instead of medial side of the kidney and aberrant ureter routing from the UPJ to the bladder (Fig. 1B). The latter phenotype appears to be secondary to the failure of *Hox10* mutant kidneys to detach from the body wall during embryogenesis (Fig. 1F and 2C and data not shown). As a result, the kidneys cannot properly rotate and ascend from the pelvic into the lumbar region as normally occurs in wild-type animals.

Lotus Tetragonolobus Lectin (LTL) and Dolichos Biflorus Agglutinin (DBA) staining in control and *Hox10* mutant kidneys sectioned through the same frontal plane further demonstrate that *Hox10* triple mutant kidneys exhibit multiple organizational defects by E16.5 (Fig. 1E, F). Normally, LTL labels the proximal tubules of mature nephrons that are confined to the cortical region, while DBA labels differentiated collecting ducts that extend radially into the medullary region (Fig. 1E). However, as depicted in Fig. 1F, both LTL and DBA staining are reduced and mislocalized in *Hox10* mutants, highlighting the significant patterning defects in these mutants.

### *Hox10* triple mutants exhibit severe ureter branching defects

Subsequent to ureteric bud invasion, the ureter begins to branch within the metanephric mesenchyme and normally undergoes six to seven branching events by E14.5 [51]. Additionally, the kidney detaches from the body wall and ascends within the body cavity. In *Hox10* mutants, the kidney fails to detach completely from the body wall and the reproductive tract (asterisks in Figure 1F, and Figure 2A, C). To compare the number of branching events and the number of early nephrons present we performed optical projection tomography (OPT) on dissected kidneys from E14.5 control and *Hox10* mutant embryos stained with pan-cytokeratin, to label the ureteric tree, and Cadherin-6 to label early nephrons (Fig. 2B, D, Movies S1 and S2, Table S1) [52]. By E14.5, *Hox10* mutant kidneys (n of 4) demonstrate a five-fold reduction in the number of ureteric branches compared to the total number of branches observed in controls (n of 3). There is also a three-fold reduction in the number of Cadherin-6-positive bodies, indicating reduced nephron formation in mutant kidneys.

### The aberrant ureter branching in *Hox10* mutants is due to intrinsic signaling defects in the developing kidney

**Figure 3:**
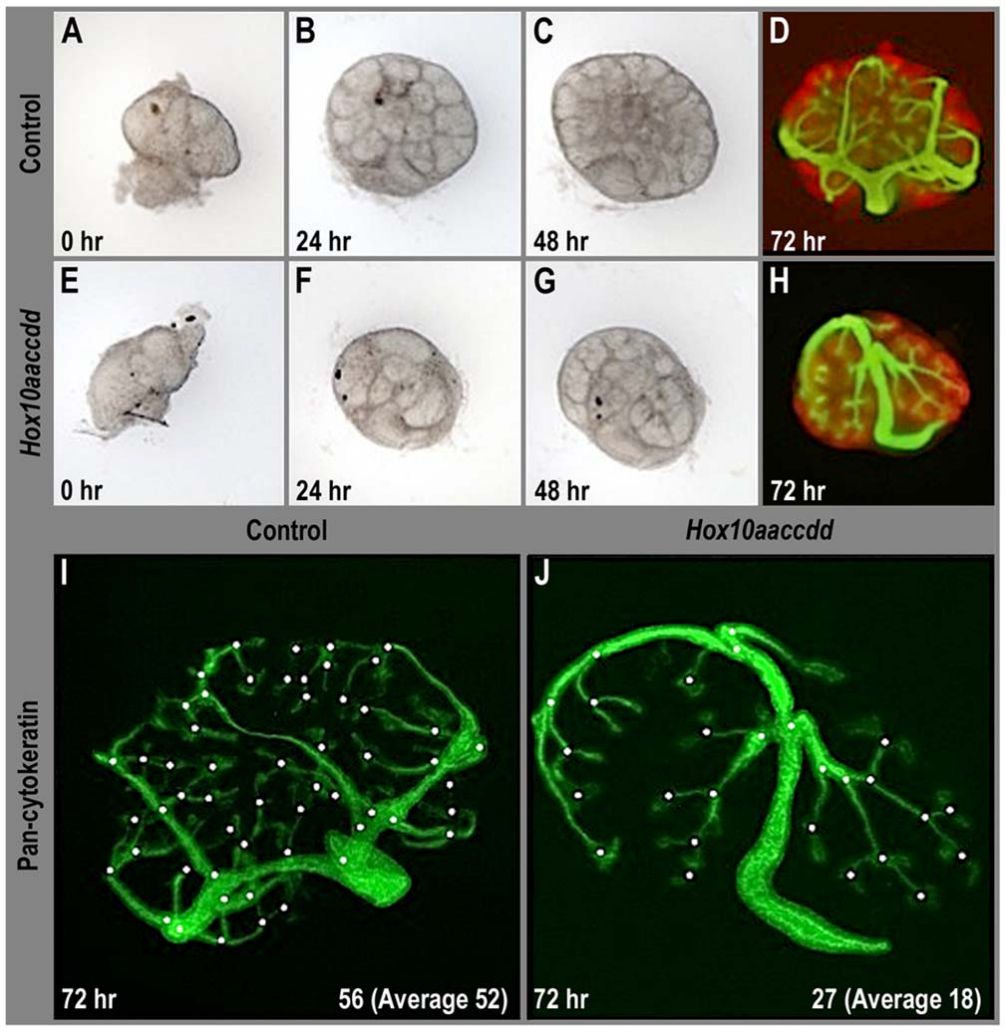
Cultured *Hox10* mutant kidneys exhibit ureter branching defects. (A–D) Control kidneys were dissected at E11.5 (A) and examined at 24 (B), 48 (C) and 72 (D) hour intervals. Pan-cytokeratin (green) was used to label the ureter epithelium and *Pax2* (red) was used to label the nephrogenic mesenchyme. (E–H) Dissected E11.5 *Hox10* mutant kidneys cultured and examined at identical time points as described for controls. Note that the *Hox10* mutant kidneys exhibit aberrant ureter branching as compared to controls. (I–J) *Hox10* mutant kidneys (J) have, on average, a 50% reduction in the number of branches as compared to controls (I).

In order to test whether the aberrant ureter branching observed in *Hox10* mutants might be secondary to its physical inability to properly detach from the body wall and ascend or intrinsic to signaling defects in the kidney, we isolated kidneys from control and *Hox10* mutant embryos at E11.5 and cultured them for 72 hours. Initially, the overall size and morphology of the tissue isolated was comparable between control and mutant kidneys (Fig 3A, E). However, the mutant kidneys were noticeably smaller and underdeveloped as compared to controls at the 24- and 48-hour time intervals (Fig 3B–C, F–G). After 72 hours in culture, the kidneys were stained for pan-cytokeratin to label the ureter epithelium and *Pax2* to label the nephrogenic mesenchyme. *Hox10* mutants develop a distinct morphological patterning defect as compared to controls, similar to what is observed *in vivo,* with increased length of each ureteric branch and less bifurcations (Fig 3D, H). *Hox10* mutants have, on average, a 50% reduction in the number of branches compared to controls (Fig 3I, J). Hence, these results support that the branching defects observed in *Hox10* mutants is due to signaling defects in the kidney and not secondary to the failure to detach from the body wall.

### *Hox10* does not effect the early expression of key nephrogenic mesenchyme markers

**Figure 4:**
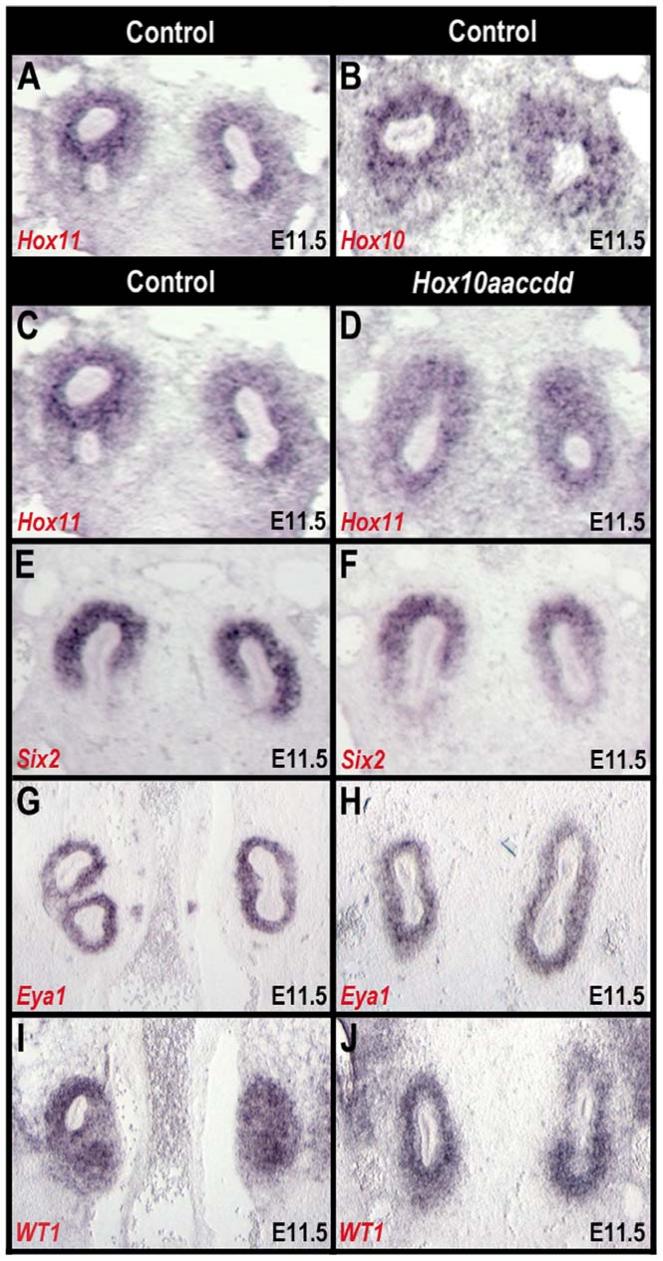
Ureteric bud formation and early expression of key nephrogenic mesenchymal markers are unaltered in *Hox10* mutants. At E11.5, both *Hox11* (A) and *Hox10* (B) are expressed in the condensed mesenchyme surrounding the ureter. (C, D) *Hox11* expression in *Hox10* mutant embryos (D) is identical to controls (C). *Six2* expression is observed similarly throughout the condensed metanephric mesenchyme of E11.5 control (E) and E11.5 mutant (F) embryos. At E11.5, the expression patterns of *Eya1* and WT1 are also unaltered in control (G, I) compared to *Hox10* mutants (H, J).

Upon ureteric bud induction, the nascent bud invades the metanephric mesenchyme ventrally. At E11.5, this step occurs indistinguishably in *Hox10* mutant animals (Fig. 4C–J). Previous studies have shown that *Hox11* genes are specifically required for the differentiation of the metanephric mesenchyme [22]. As depicted in Fig. 4A–B, *Hox10* and *Hox11* are both expressed in the metanephric mesenchyme surrounding the ureter at E11.5. This overlap of expression domains suggests that *Hox10* could also play a role in the early patterning of the nephrogenic mesenchyme [18], [22], [42]. However, the expression patterns of *Hox11*, *Six2*, *Wt1* and *Eya1,* all transcription factors that are important for early nephrogenic mesenchyme events [18], [19], [22], [23], [29], [53], are unaffected in *Hox10* triple mutants and remain strongly expressed throughout the condensed nephrogenic mesenchyme (Figs 4C–J). In addition, the fact that the expression patterns of these genes are all unaffected indicates that the nephrogenic mesenchyme, a zone of critical importance for the initial events of kidney development, is both properly established and maintained through E11.5 in *Hox10* triple mutants. Therefore, despite the clear overlap of expression (Fig 4A–B), these data demonstrate no defects in the early metanephric mesenchyme and suggest a unique function for *Hox10* paralogous group genes in kidney organogenesis.

### Expression of *Hox10* and *Hox11* in the developing kidney

**Figure 5:**
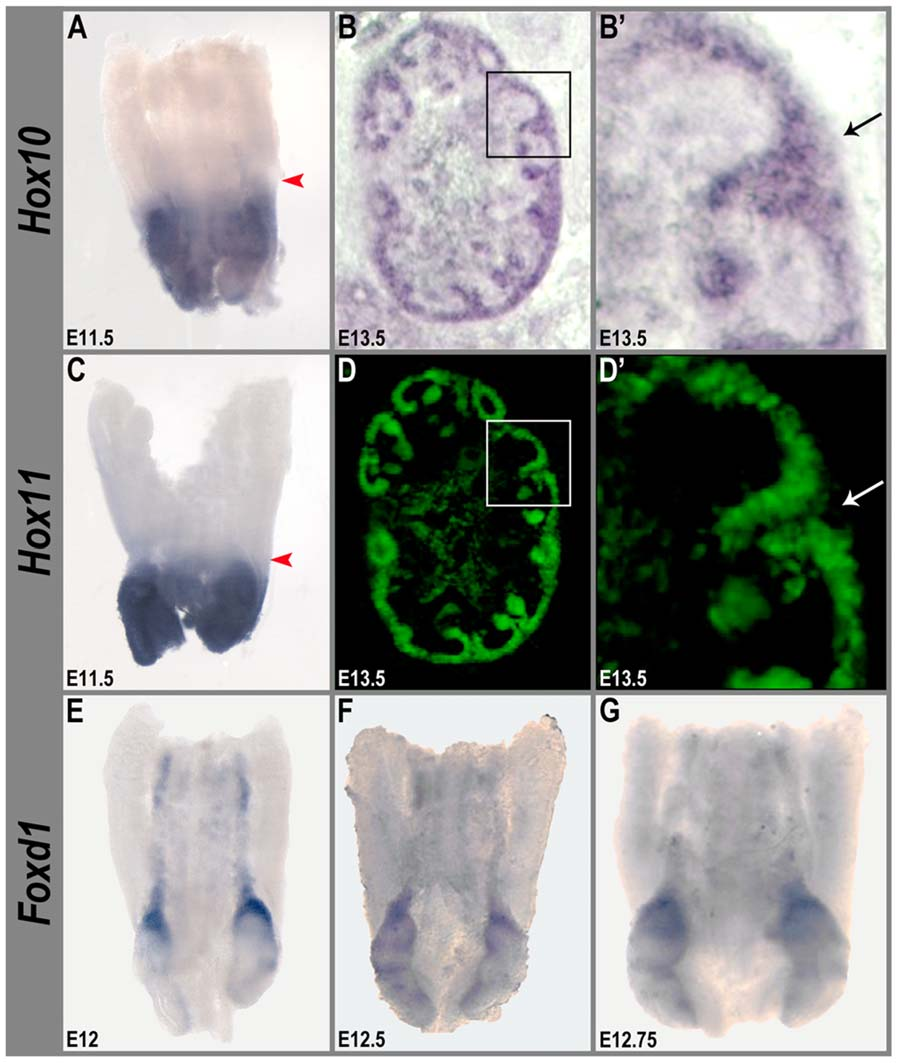
Expression patterns of *Hox10, Hox11* and *Foxd1* in the early nephrogenic cord. At E11.5, the *Hox10* genes (A) exhibit a more anterior boundary of expression in dissected urogentital mesenchyme compared to *Hox11* in control mice (Compare arrowheads in A and C). By E13.5, *Hox10* (B) and *Hoxa11*eGFP (D) are both expressed in the nephrogenic cap mesenchyme, but *Hox10* genes (B') are additionally expressed in the cortical stroma cells (black arrow), whereas *Hox11* (D') is expressed in the renal vesicles but not in the cortical stroma (white arrow). (E) At E11.5, *Foxd1* expression forms a highly concentrated cap-like pattern localized just anterior to the metanephric kidney. (F–G) As development progresses, these *Foxd1*-expressing cells are observed in progressively posterior positions (F, G). Panels (B–B') and (D–D') show light microscopy and fluorescent images of the same section, respectively. In situ hybridization analyses for *Hox10* was performed using probes to all three paralogs (*Hoxa10*, *Hoxc10*, *Hoxd10*).

To obtain a better understanding into the differences between *Hox10* and *Hox11* function in the kidney, we performed detailed expression analyses on both genes during multiple stages of development and carefully compared the two patterns. As previously described, *in-situ* hybridization analyses performed on tissue sections from E11.5 show that both *Hox10* and *Hox11* are expressed in the metanephric mesenchyme surrounding the ureter in similar patterns (Fig. 4A–B). However, *in-situ* hybridization experiments performed on dissected urogenital tissue from the same stage show that the anterior borders of *Hox10* and *Hox11* expression are clearly different (Fig. 5A, C arrowheads). As shown in Fig. 5C, the anterior limit of *Hox11* expression is at the anterior border of the metanephric mesenchyme. In contrast, *Hox10* expression is not limited to the metanephric mesenchyme, but extends into a more anterior region of the nephrogenic cord (Fig. 5A arrowhead). Thus, there are fundamental differences in the expression patterns of *Hox10* and *Hox11* genes even at these early stages of kidney organogenesis.

In order to directly compare *Hox10* and *Hox11* expression patterns at later developmental stages, we performed *Hox10* in situ hybridization analyses on tissue sections from a *Hoxa11*eGFP reporter line in which GFP fluorescence has been shown to accurately recapitulate *Hox11* expression ([43], Figure S1). At E13.5, both *Hox10* and *Hox11* are expressed in the nephrogenic mesenchyme (Fig. 5B, B', D, D'). However, *Hox10* genes exhibit additional, unique expression in the thin layer of cells surrounding the nephrogenic mesenchyme known as the cortical stromal cells (Compare arrows in Fig. 5B' to 5D'). Thus, *Hox10* genes are expressed in both the stromal and nephrogenic mesenchyme compartments of the kidney at this stage, whereas the *Hox11* genes are expressed exclusively in the nephrogenic mesenchyme. In addition, the difference in expression domains is consistent with the possibility that *Hox10* genes play a unique role in cortical stromal cell development.

### *Hox10* is required for cortical stromal cell function

**Figure 6:**
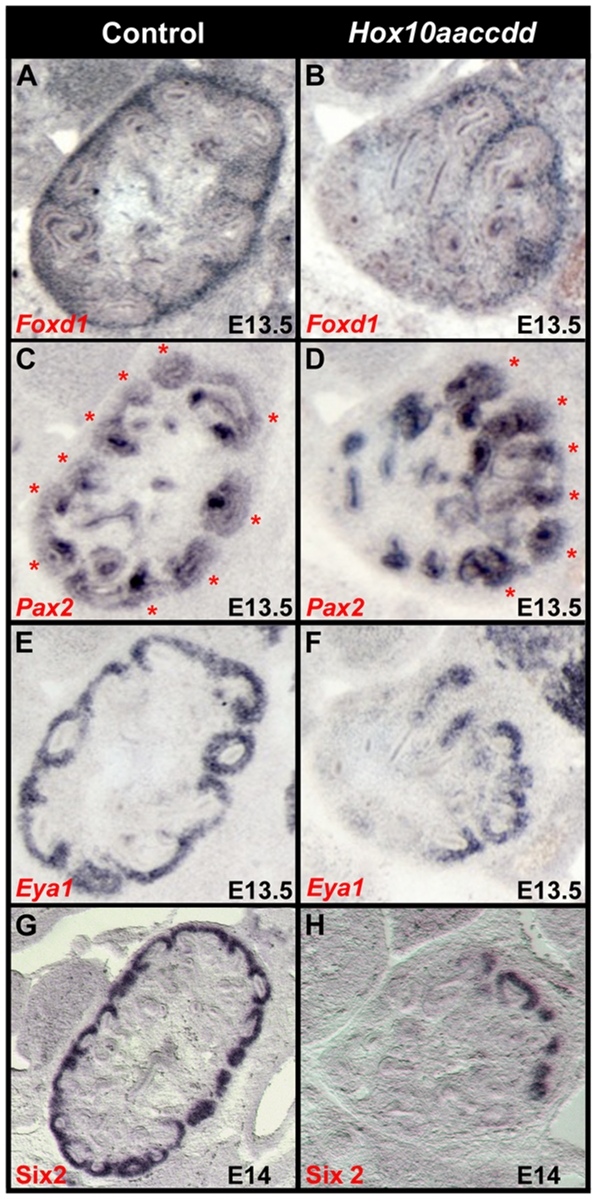
Nephrogenic mesenchyme markers become restricted to regions of *Foxd1* cell integration. (A–F) Serial sections through control (A, C, E) and *Hox10* mutants (B, D, F) at E13.5. (A) *Foxd1* expression is observed in the cortical stromal cells fully integrated into the periphery of the kidney at E13.5 in controls. (B) *Foxd1* signal is regionally restricted in *Hox10* mutants. (C–D) Expression of the nephrogenic marker *Pax2* in control (C) and *Hox10* mutant (D) embryos. In *Hox10* mutants, *Pax2* (D) is restricted to the domain in the kidney that is selectively expressing *Foxd1*. Red asterisks depict nephrogenic mesenchymal expression of *Pax2* in (C, D). Similar patterns are observed with both *Eya1*(E, F) and *Six2* (G,H) in which the normal expression in the mesenchymal condensations in the in the nephrogenic zone (E, G) becomes positionally restricted to regions of *Foxd1*-expressing cells in the *Hox10* mutant (F, H).

Previous studies have shown that the stromal cell population in the developing kidney is critical for proper branching and differentiation of the kidney capsule [35], [36]. The earliest known marker for this cell lineage is the transcription factor *Foxd1*, which is exclusively expressed in the cortical stromal cells as early as E11.5 [34]. Consistent with these data, whole mount in situ hybridization analyses performed on dissected urogenital tissue show that *Foxd1* cannot be detected until E11.5. At E11.5, *Foxd1*-expressing cells are observed in a highly concentrated cap-like pattern that is localized just anterior to the metanephric mesenchyme (Fig 5E). Over approximately the next 12 hours of development, *Foxd1*-expressing cells are observed in progressively posterior positions, (Fig. 5F, G), and by E13.5, the cortical stromal cells surround and are fully integrated into the developing kidney (Fig. 6A). While *Foxd1*-expressing cells initiate normally in *Hox10* mutants, they do not fully integrate into the kidney in *Hox10* mutants (Fig. 6B). Instead, they are observed only in a restricted subset of cells at the periphery of the kidney (Figs. 6A, B). These data demonstrate that *Hox10* has a critical function in promoting the proper integration of these cells into the developing kidney.

### Nephrogenic mesenchyme markers become restricted to regions where *Foxd1-*expressing cells integrate

We next examined whether the restricted expression domain of *Foxd1* in the periphery of the developing kidney in *Hox10* mutants might lead to a localized signaling environment in which proper UB branching and nephrogenesis are also restricted. While the ureteric epithelial expression of *Pax2* remains intact at E13.5 in *Hox10* triple mutants, *Pax2* expression in the nephrogenic mesenchyme becomes restricted to the domain in the periphery of the kidney adjacent to the remaining *Foxd1*-expressing cells (Fig. 6C, D asterisks). Similarly, the expression of the nephrogenic markers *Eya1* and *Six2* also become restricted to the region immediately adjacent to the *Foxd1*-expressing cells in the kidney periphery in *Hox10* mutants (Fig. 6F, H). These results imply that the continued expressions of these key nephrogenic mesenchymal markers in *Hox10* mutants are both dependent upon and reacting to the proper integration of stromal cells.

**Figure 7:**
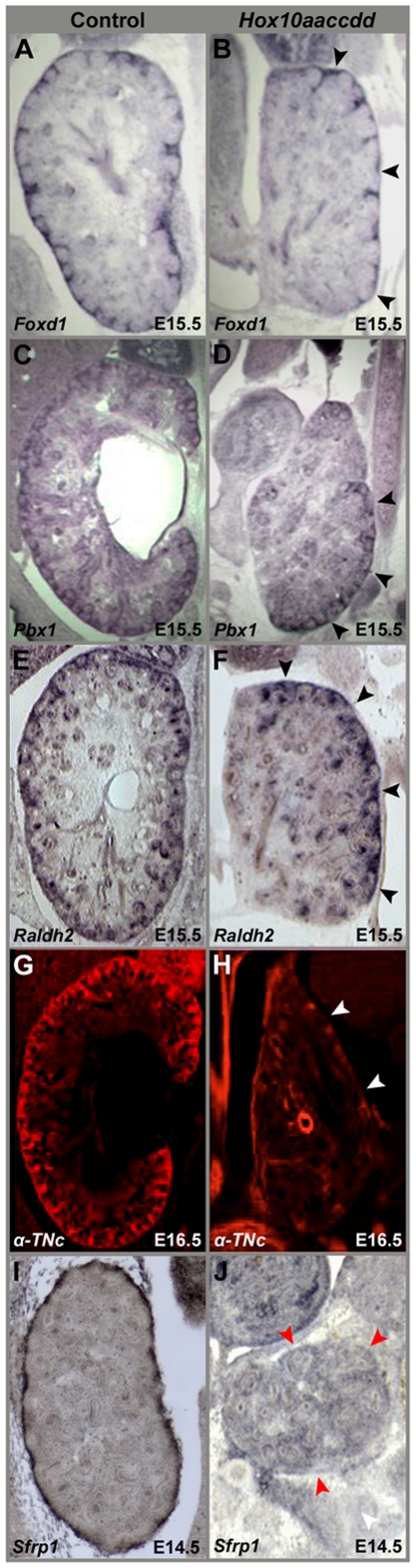
Cortical stromal cells fail to properly differentiate and the kidney capsule does not form in *Hox10* mutants. (A) In control embryos, *Foxd1* is expressed in the cortical stromal cells that surround the kidney at E15.5. (B) *Foxd1* signal in *Hox10* mutants is restricted peripherally in the developing kidney (arrowheads). (C–F) *Pbx1* and *Raldh2* are known markers for stromal cell differentiation that are normally expressed in the nephrogenic zone and cortical stroma of the developing kidney in control embryos (C, E). The expression of both of these genes is restricted (D, F arrowheads) in a pattern similar to what is observed for *Foxd1* in *Hox10* mutants (B). (G) Tenascin-C is expressed in differentiated cortical stromal cells in controls. (H) Tenascin-C expression is lost in the kidney periphery in *Hox10* mutants (arrowheads). (I–J) *Sfrp1* expression in control (I) and *Hox10* mutant (J) E14.5 embryos. *Sfrp1* is strongly expressed in the kidney capsule of control animals (J) and is missing in the periphery (arrowheads) of *Hox10* mutant kidneys (J).

We next examined the expression patterns of several genes that have reported functions in cortical cell differentiation in both control and *Hox10* triple mutant kidneys. At E15.5, *Foxd1* expression continues to be restricted in the developing kidney in *Hox10* mutants (Fig. 7A, B). Other important markers for stromal cell differentiation include *Pbx1* and *Raldh2* [39], [54], [55], [56]. Normally, these stromal markers are also expressed around the entire periphery in the developing kidney at E15.5 (Fig. 7C, E). However, in *Hox10* mutant kidneys, the expression patterns of both of these markers become restricted to the same regions in the periphery of the kidney as *Foxd1* (Fig 7D, F). These data provide further evidence that *Hox10* plays an important role in the proper integration of the cortical stromal cells during kidney organogenesis.

### Kidney capsule maturation is disrupted in *Hox10* mutants

Tenascin-C is a marker for differentiated stromal cells and is normally detected in the kidney periphery within the population of cells that has been postulated to play a role in renal capsule formation (Fig 7G; [34]. In contrast to the peripheral expression of Tenascin-C in differentiated cortical stromal cells in control embryos, this signal is completely lost in *Hox10* mutants and indicates that this population of cells fails to properly differentiate (Fig. 7G, H). In addition to Tenascin-C, previous studies have shown that the secreted frizzled-related protein *Sfrp1* is strongly expressed in the renal capsule [36], [57], [58], [59]. Consistent with these reports, we also find that *Sfrp1* is expressed at a high level throughout the kidney capsule cell layer and surrounds the periphery of the developing kidney at E14.5 in control embryos (Fig. 7I). However, there is a complete down-regulation of this gene in *Hox10* mutants (Fig. 7J arrowheads). Thus, it appears that *Hox10* plays in role in the regulation of kidney capsule formation that is similar to what has recently reported for *Foxd1* [36].

**Figure 8:**
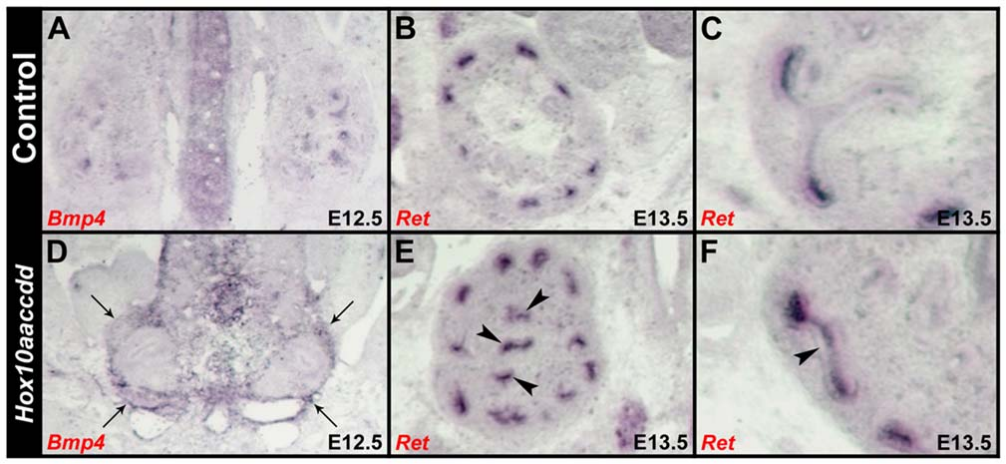
*Hox10* kidneys demonstrate aberrant branching defects and are exposed to elevated levels of *Bmp4*. (A, D) *Bmp4* is normally down-regulated in the body wall around the kidney by E12.5 (A), but this expression is ectopically maintained in *Hox10* mutants (D). (B, C) *Ret* is normally expressed in the tips of the UB, providing an inductive signal that stimulates the growth of new ureter branches. (E, F) Ectopic *Ret* expression is observed in extended regions along the stalks of the ampulla (arrowheads) in *Hox10* mutants.

A previous study has shown elevated ectopic levels of *Bmp4* can inhibit the proper formation of the kidney capsule [36]. Normally, *Bmp4* expression is confined to interior regions of the maturing kidney where it inhibits nephron differentiation [36], [60], [61]. Consistent with the previous studies and loss of kidney capsule in these mutants, *Hox10* mutants are also exposed to elevated levels of *Bmp4* expression surrounding the mutant kidney (Compare Figs. 8A, D). Hence, our data suggest that the improper formation of the kidney capsule in *Hox10* mutants may be due in part to the exposure of ectopic elevated levels of *Bmp4*.

The loss of proper cortical stromal cell differentiation leads to an improper signaling environment that can secondarily affect ureter branching. Normally, *Ret* expression is localized in the nephron progenitors at the tips of the UB and provides an inductive signal that stimulates the growth of new ureter branches (Fig. 8B, C; [36]). However, in *Hox10* mutants, *Ret* expression is not solely localized to the UB tips and instead, can be observed in extended regions along the stalks of the nascent ampulla (Fig. 8E, F arrowheads), consistent with the observed branching defects in *Hox10* mutant kidneys.

## Discussion

**Figure 9:**
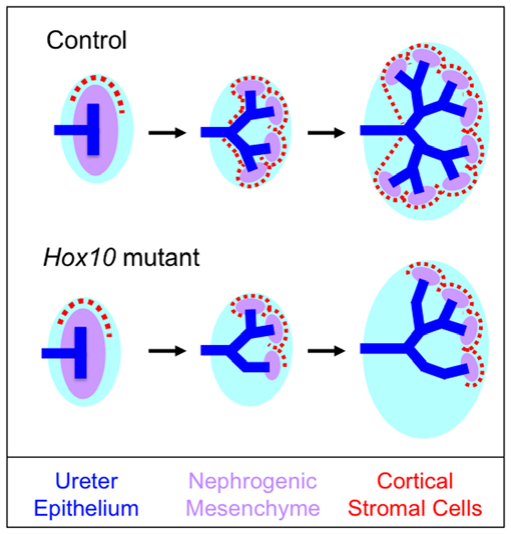
Model depicting the role of the *Hox10* genes in the proper differentiation and integration of the cortical stromal cells during mammalian kidney development. In control animals, the cortical stromal cells are initially concentrated in a cap-like region just anterior to the metanephric kidney. As development progresses, these cells become integrated into the periphery of the kidney. Once integrated, the cortical stromal cells promote proper ureter branching and nephron differentiation. In *Hox10* mutants, while the formation of the initial cortical stromal population is unaffected, these cells fail to properly integrate into the kidney periphery. The absence of cortical stromal cells in parts of the developing kidney creates distinct areas of signaling defects that secondarily results in aberrant ureter branching and decreased nephrogenesis.

Interestingly, *Hox10* and *Hox11* mutant mice yield distinct kidney phenotypes despite largely overlapping expression patterns throughout kidney development. Previous studies have shown that the *Hox11* genes play a critical role in the formation and invasion of the UB via the upregulation of *Gdnf* and *Six2* in the metanephric mesenchyme [18], [22]. As a result of the loss of *Gdnf*, kidneys do not form in *Hox11* mutant mice. In contrast, the initial stages of kidney development are unaffected in *Hox10* triple mutants. These mice undergo normal UB induction, and initiate the proper expression of several key transcription factors essential for the formation, proliferation and survival of the nephrogenic mesenchyme. These data indicate that, unlike the situation observed in *Hox11* mutants, the nephrogenic mesenchyme is properly formed in *Hox10* triple mutants. It is not until later stages of organogenesis that the primary defect of *Hox10* triple mutants is observed. The cortical stromal cells fail to properly differentiate and integrate into the developing kidney. The defect in cortical stromal cell development in *Hox10* mutants leads to additional phenotypes including aberrant ureter branching and decreased nephrogenesis (Fig. 9).

Further support for a role for *Hox10* genes in cortical stromal cell function comes from the fact that many of the structural and molecular defects in *Hox10* mutants are highly reminiscent of those that have been reported for *Foxd1*. Specifically, loss of *Foxd1* function results in hypoplastic kidneys, impaired branching morphogenesis and reduction of nephron number [34], [36]. In addition, *Foxd1* mutant kidneys fail to detach from the body wall during embryogenesis and as a result, do not ascend from the pelvic to lumbar region. All of these characteristics are phenocopied in *Hox10* triple mutants and therefore provide further support for *Hox10* genes playing a critical role in the development and integration of cortical stromal cells. It is interesting to note however, that the initial stromal cell precursor population is retained in both *Foxd1* and *Hox10* mutants. Additionally, we show that *Hox10* does not regulate *Foxd1* expression in the cortical stromal cells. Thus, *Hox10* does not regulate the initial formation of the cortical stromal cell population, but rather, governs the proper integration and subsequent differentiation of this lineage in the developing metanephric kidney.

A previous study has shown that the cortical stromal cells play an important role in regulating the formation of the kidney capsule [36]. Both *Hox10* and *Foxd1* mutants demonstrate defects in capsule development, as evidenced by the loss *Sfrp1* and Tenascin-C expression in the periphery of the developing kidney [34], [36]. Discerning the mechanisms by which *Hox10* genes regulate the differentiation and integration of this population will be the focus of future studies.

Another key question regards the cellular origins of the cortical stromal cell lineage. Previous studies have suggested that stromal cells may be derived from the metanephric blastema, either as cells that do not undergo nephron differentiation like the cap mesenchyme, or from a unique cell lineage present in the blastema [32], [62], [63]. More recently, a lineage trace with an *Osr1*-Cre provided evidence that the initial population of *Foxd1-*expressing cells are derived from the intermediate mesoderm [64]. Alternatively, a recent fate map study in chick suggests that the kidney stromal cells may be derived from paraxial mesoderm progenitors in this species [65]. We report here that *Foxd1*-expressing cells are initially observed as a highly concentrated cap-like pattern that is localized just anterior to the metanephric mesenchyme. As development progresses, these cells are observed in progressively posterior positions, becoming integrated into the periphery of the kidney to form the cortical stroma. These data are consistent with the cortical stromal cell lineage arising from the nephrogenic mesenchyme in a region just anterior to the metanephric mesenchyme. The more anterior border of *Hox10* expression as compared to *Hox11* is consistent with a unique role for *Hox10* in the formation of this cell type. In support of this hypothesis, Mugford et al (2008b) showed that while the stromal cell lineage was labeled strongly when *Osr1*-Cre was activated at early developmental stages, while only the nephrogenic mesenchyme and not the cortical stroma was labeled when *Osr1*-Cre was activated at later stages. This result provides additional support for our hypothesis that the cortical stromal cell lineage arises from a population of cells that is anterior to the metanephric mesenchyme. Hence, the combined data suggest that the general AP patterning functions of *Hox* genes directly translates into the differentiation of the distinct cell populations in the kidney. Future experiments removing *Hox10* (or *Foxd1*) in specific cell populations will allow for a more definitive determination of when and where these early regulatory signals are required for the appropriate differentiation and integration of the kidney cortical stroma cells.

## Materials and Methods

### Animals and histology

Generation of *Hox10* mutant embryos was previously described [45]. Embryos and kidneys were dissected in PBS, fixed in formalin for one to three hours, and dehydrated through graded alcohols and stored in 70% ethanol at 4°C. Embryos were vacuum-embedded in paraffin, sectioned at 5 µm and stained with hematoxylin and eosin. A piece of tissue from the embryo was used for genotyping. All animal experiments performed in this report were reviewed and approved by the University of Michigan's Committee on Use and Care of Animals, Protocol #08787, under Animal Welfare Assurance #A3114-01 on file with the NIH Office of Laboratory Animal Welfare.

### *In situ* hybridization

Whole mount *in situ* hybridization was performed as previously described [22], [66].

For section *in situ* hybridization, embryos were collected in PBS and fixed overnight in 4% paraformaldehyde in PBS (PFA) at 4°C. Embryos were then rinsed in PBS and immersed in 30% sucrose at 4°C overnight prior to embedding into OCT media. 20 to 30 µm frozen sections were cut and slides were stored at −80°C.

*In situ* hybridization (ISH) was performed as previously described [37], [67]. Prior to ISH on sections of *Hoxa11eGFP* tissue, slides were rinsed in PBS and fluorescent images were taken on an Olympus BX-51 upright light microscope with an Olympus DP70 camera. *Hoxa10*, *Hoxc10*, and *Hoxd10 in situ* probes were previously described [68], [69], [70].

### Immunohistochemistry (IHC)

For IHC on sections, embryos were processed and sectioned as described above for section ISH. IHC using antibodies to E-cadherin (R&D Systems) and smooth muscle actin (Cy3-conjugated mouse monoclonal, Sigma) were performed as previously described [71]. Anti-E-cadherin was used in a dilution of 1∶100 and detected by a 1∶500 dilution of donkey anti-goat Alexafluor 488 (Invitrogen). Anit-smooth muscle actin was used in a dilution of 1∶400. Immunohistochemical (IHC) localization of pan-cytokeratin (Sigma) on whole embryonic kidneys at E12.5 and E13.5 was performed as previously described [17], [52]. Anti-pan-cytokeratin was used in a dilution of 1∶200 and detected by a 1∶300 dilution of goat anti-mouse FITC (Jackson Immunolabs).

Lectin immunohistochemistry was performed as previously described [37]. After washing in PBS, slides were incubated in 50 mM NH_4_Cl in PBS at room temperature for 20 minutes, followed by a 20 minute incubation with GSP solution (0.2% gelatin+0.075% saponin (S4521, Sigma) in PBS) at 37°C. Slides were then incubated with 1∶250 rhodamine labeled DBA (Vector Labs) and 1∶400 fluorescein labeled LTA (Vector Labs) at 4°C overnight in the humidifying chamber. Slides were then incubated with GSP solution at 37°C two times for 20 minutes and rinsed in PBS+0.05% tween at room temperature. After washing for 20 minutes with PBS, slides were mounted with Pro-Long Gold, antifade reagent (Invitrogen).

### OPT imaging of embryonic kidneys

E14.5 kidneys were collected in ice cold PBS and fixed in 4% PFA for 10 minutes. They were washed two times in cold PBS and then washed in TBS for 20 minutes. After washing the kidneys in TBS+1% triton (TBSTr) for 20 minutes, they were blocked in TBSTr+1% BSA at room temperature for one hour. They were then incubated overnight at 4°C rocking with anti-pan-cytokeratin (1∶100) and anti-Cadherin-6 (1∶200) in TBSTr+1% donkey serum. The next day they were washed in TBSTr three to four times at room temperature and one time overnight at 4°C. Kidneys were then incubated overnight at 4°C rocking with donkey anti-mouse Alexafluor 555 (Invitrogen, 1∶100) and goat anti-rabbit Alexafluor 488 (Invitrogen, 1∶200) diluted in TBSTr+1% donkey serum. After washing four to five times at room temperature and one time overnight at 4°C in TBSTr, the tissue was fixed for 15 minutes in 4% PFA and washed three times 10 minutes in TBS. Kidneys were then stored in TBS+0.1% sodium azide prior to embedding.

Stained kidneys were embedded in warm 1% low melting point agarose and left until set. Slices containing the specimen were excised, and glued to aluminium-magnetic mounts. Specimens were then dehydrated in 100% methanol for 6 hours with 3 changes of methanol, and then cleared overnight in Benzyl Alcohol Benzyl Benzoate mixed at a ratio of 1∶2. Once clear, samples were imaged in a Bioptonics 3001 OPT scanner (Bioptonics, UK), at maximum resolution of 3.2 µm per pixel zoom. Images were acquired at 0.9 degree intervals, with each frame averaged over 4 images. Quantification/Skeletonisation was done using a Kidney Analysis Application [72]. Rendering/Visualization was performed using Drishti http://anusf.anu.edu.au/Vizlab/drishti/.

## Supporting Information

Figure S1 ***Hox11*** **mRNA expression overlaps with Hoxa11eGFP reporter expression.** *Hox11 in situ* analysis (A) was done on tissue sections from an E13.5 *Hoxa11eGFP* heterozygous embryo (B). *Hox11* mRNA and Hoxa11eGFP are both expressed in the nephrogenic cap mesenchyme (white arrows in A' and B') and not in the cortical stroma cells.
(TIF)

Movie S1 Movie rendering of OPT analysis of a control kidney, immunostained with pan-cytokeratin (red) and cadherin 6 (green). The brown color represents unstained space filling of the rest of the dissected tissue.
(MOV)

Movie S2 Movie rendering of OPT analysis of two *Hox10* triple mutant kidneys, immunostained with pan-cytokeratin (red) and cadherin 6 (green). The brown color represents unstained space filling of the rest of the dissected tissue. Of note, in mutant kidneys, some ureteric tips are not associated with nephrogenic mesenchyme.
(MOV)

Table S1 **Ureteric Tree Branch Analysis.**
(DOCX)
