# Supplementary figures and images for: Hox10 Genes Function in Kidney Development in the Differentiation and Integration of the Cortical Stroma
Source: PLoS One. 2011 Aug 16;6(8):e23410. doi: 10.1371/journal.pone.0023410 (PMC3156768; doi:10.1371/journal.pone.0023410)

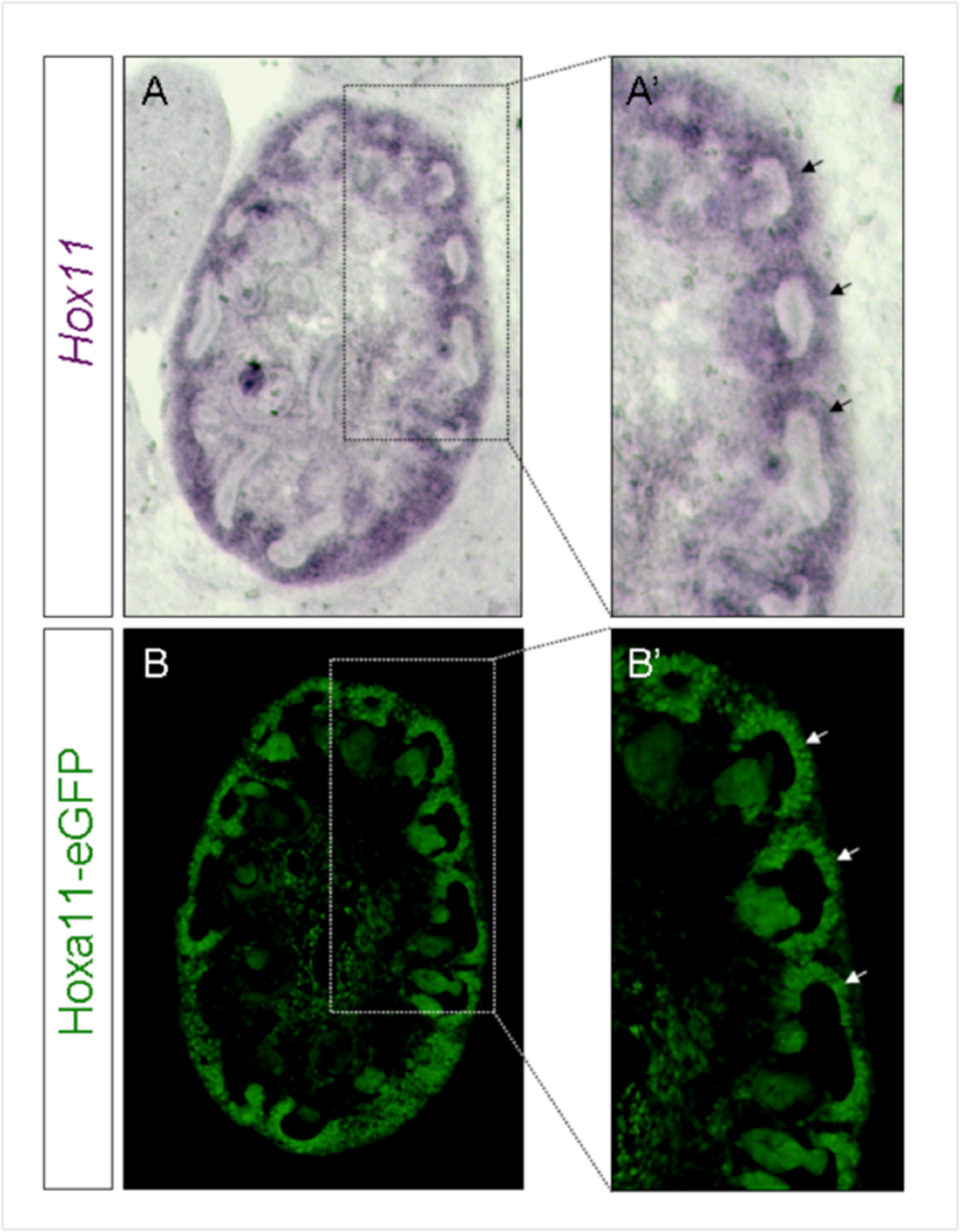

Supplement: Figure S1 — Hox11 mRNA expression overlaps with Hoxa11eGFP reporter expression. Hox11 in situ analysis (A) was done on tissue sections from an E13.5 Hoxa11eGFP heterozygous embryo (B). Hox11 mRNA and Hoxa11eGFP are both expressed in the nephrogenic cap mesenchyme (white arrows in A' and B') and not in the cortical stroma cells. (TIF) [file pone.0023410.s001.tif]
