# Supplementary material for: Hox10 Genes Function in Kidney Development in the Differentiation and Integration of the Cortical Stroma
Source: PLoS One. 2011 Aug 16;6(8):e23410. doi: 10.1371/journal.pone.0023410 (PMC3156768; doi:10.1371/journal.pone.0023410)
Supplement: Table S1 — Ureteric Tree Branch Analysis. (DOCX) [file pone.0023410.s004.docx]

**Table S1:**

| **Supplemental Table 1.**  **Ureteric Tree Branch Analysis** | |  |
| --- | --- | --- |
|  | Branched skeletal voxels | Cad6+ bodies |
| Control 1 - left kidney | 78 | 52 |
| Control 2 - left kidney | 166 |  |
| Control 2 - right kidney | 143 |  |
| **Average** | **129** | **52** |
|  |  |  |
| Mutant 1 - left kidney | 11 | 18 |
| Mutant 1 - right kidney | 10 | 17 |
| Mutant 2 - left kidney | 44 |  |
| Mutant 2 - right kidney | 32 |  |
| **Average** | **24.25** | **17.5** |
